# Crystal structure of (perchlorato-κ*O*)(1,4,7,10-tetra­aza­cyclo­dodecane-κ^4^ *N*)copper(II) perchlorate

**DOI:** 10.1107/S2056989016019563

**Published:** 2017-01-01

**Authors:** Jessica L. Gray, Deidra L. Gerlach, Elizabeth T. Papish

**Affiliations:** a Department of Chemistry, University of Alabama, 250 Hackberry Lane, Tuscaloosa, AL 35487-0336, USA

**Keywords:** crystal structure, copper(II), cyclen, perchlorate

## Abstract

The crystal structure of the title salt, [Cu(ClO_4_)(C_8_H_20_N_4_)]ClO_4_, is reported. The Cu^II^ ion exhibits a square-pyramidal geometry and is coordinated by the four N atoms of the neutral 1,4,7,10-tetra­aza­cyclo­dodecane (cyclen) ligand and an O atom from one perchlorate anion, with the second perchlorate ion hydrogen-bonded to one of the amine N atoms of the cyclen ligand. Additional N—H⋯O hydrogen bonds between the amine H atoms and the coordinating and non-coordinating perchlorate groups create a three-dimensional network structure. Crystals were grown from a concentrated methanol solution at ambient temperature, resulting in no co-crystallization of solvent.

## Abstract 2

The crystal structure of (perchlorato-κ*O*)(1,4,7,10-tetra­aza­cyclo­dodecane-κ^4^ *N*)copper(II) perchlorate is reported. The crystal was grown from a solution of methanol at ambient temperature which resulted in no co-crystallization of solvent.

## Chemical context   

Aza­macrocycle ligands, including 1,4,7,10-tetra­aza­cyclo­dodecane (cyclen), are of significant importance in research due to their ability to form stable metal complexes, allowing for their use in a wide range of applications. Some of these complexes have been studied for their use as chemical sensors, contrast agents in MRI and PET, anti­microbial agents and as biomimetic catalysts (De León-Rodríguez *et al.*, 2010 ▸; Yoo *et al.*, 2005 ▸). Copper–cyclen complexes have been studied extensively for their ability to perform catalytic DNA cleavage and peptide hydrolysis (Zhang *et al.*, 2016 ▸; Li *et al.* 2014 ▸; Hormann *et al.*, 2015 ▸). Although the synthesis of a similar Cu^II^ complex has been reported previously, no crystal structure of the complex, [Cu(1,4,7,10-tetra­aza­cyclo­dodeca­ne)](ClO_4_)_2_, has previously been published (Kruppa *et al.*, 2006 ▸).
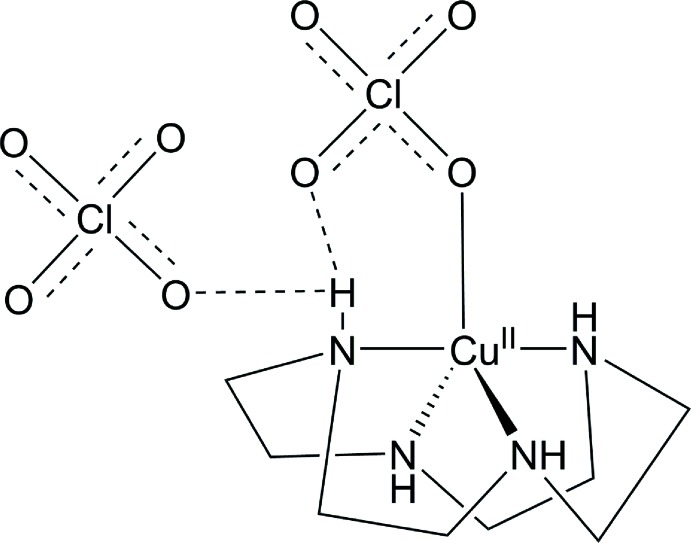


## Structural commentary   

**Figure 1:**
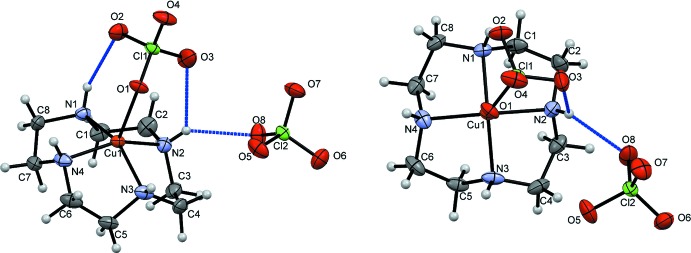
Side (left) and top (right) views, as defined by the cyclen ligand ring, of [Cu(cyclen)](ClO_4_)_2_ represented with ellipsoids at the 50% probability level. Hydrogen bonds are drawn in blue.

In the title complex (Fig. 1 ▸), the copper(II) ion coordinated by the four nitro­gen atoms of the cyclen ligand and one oxygen atom of a perchlorate ligand. The five-coordinate cupric ion shows a nearly ideal square-pyramidal geometry (τ_5_ = 0.049; Addison *et al.*, 1984 ▸). The Cu—N bond lengths range from 2.004 (1) to 2.015 (1) Å, which are typical values. The Cu^II^ ion exhibits a tetra­gonal distortion that leads to a longer apical bond with Cu1—O1 = 2.266 (1) Å, which is 0.12 Å longer than the average Cu—O distance (Clay *et al.*, 1979 ▸; Rohde & Merzweiler, 2010 ▸). The average N—Cu—O bond angle is 103.8 (8)°. Three hydrogen bonds are present within the asymmetric unit, with two extending from O2 and O3 of the bound perchlorate anion to N1—H1 and N2—H2, respectively. The third hydrogen bond extends from N2—H2 to O8 of the unbound anion; the numerical details are given in Table 1 ▸.

## Supra­molecular features   

**Figure 2:**
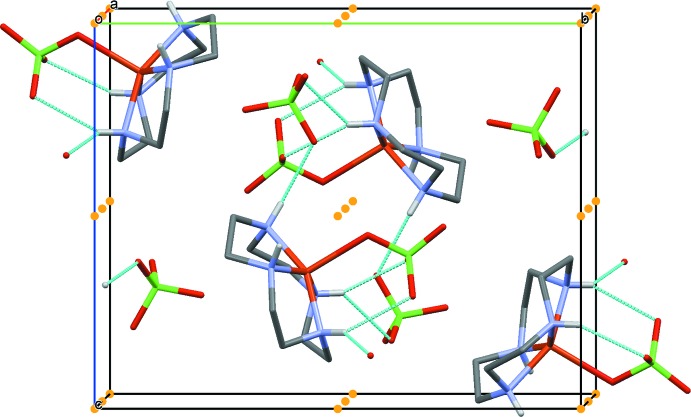
View of the unit cell along the *a* axis. An inversion center (yellow dots) exists between two asymmetric units, creating the dimeric unit defined at the center of the unit cell. Hydrogen bonds are drawn in blue.

**Figure 3:**
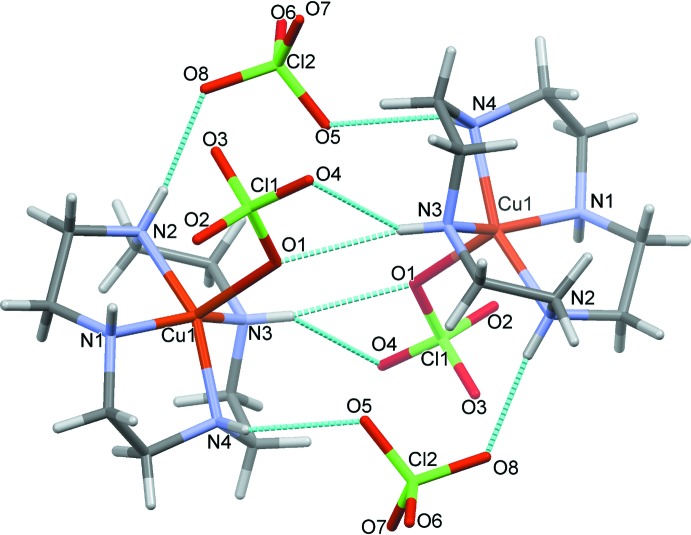
A view of hydrogen bonding within a dimer pair. Hydrogen bonds are drawn in blue. Carbon and hydrogen atom labels have been omitted for clarity.

**Figure 4:**
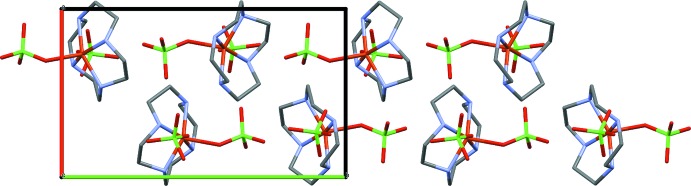
Packing of the complex cations, as viewed along the *c* axis of the unit cell. The *a* axis is drawn in red and the *b* axis is drawn in green.

**Figure 5:**
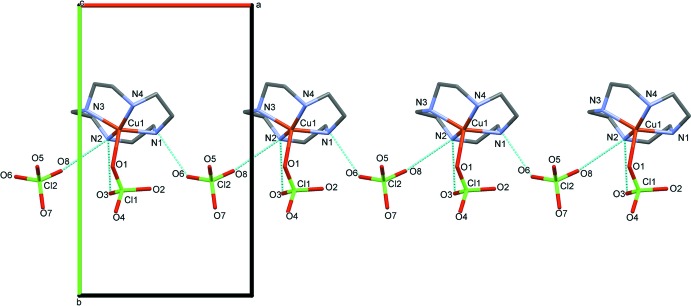
Hydrogen bonding between complex cations and anions, as viewed along the *c* axis. Hydrogen bonds are drawn in blue. The *a* axis is drawn in red and the *b* axis is drawn in green.

The crystal structure exhibits three unique symmetry elements: an inversion center, a twofold screw axis and a glide plane. The complex cations of two asymmetric units hydrogen-bond across an inversion center, which is clearly visible when viewed along the *a* axis (Fig. 2 ▸), creating a dimer. These hydrogen bonds (N3—H3⋯O1, N3—H3⋯O4, N4—H4⋯O5) have an average N⋯O distance of 3.16 Å (Fig. 3 ▸). The complexes assemble in rows parallel to the *b* axis (Fig. 4 ▸) due in part to weak electrostatic inter­actions between the bound perchlorate anion and a neighboring cyclen ligand. A hydrogen bond between the cyclen ligand and a neighboring perchlorate anion (N1—H1⋯O3) allows the building units to assemble parallel to the *a* axis (Fig. 5 ▸).

## Database survey   

A database survey resulted in several similar Cu–cyclen complexes with five-coordinate copper(II). Four structures chosen for further analysis contained a copper(II) ion coordinated by either five nitro­gen atoms or four nitro­gen atoms and one oxygen atom (Rohde & Merzweiler, 2010 ▸; Sarma *et al.*, 2010 ▸; Péréz-Toro *et al.*, 2015 ▸; Guo *et al.*, 2008 ▸). Where applicable, the complexes have similar Cu—O bond lengths to that of the title complex, with only slight deviations. The title complex and surveyed complexes have similar Cu—N distances with a standard deviation of 0.018 Å.

## Synthesis and crystallization   

The title complex was synthesized by a modified method as reported by Kruppa *et al.* (2006 ▸). Under a nitro­gen atmos­phere, 1,4,7,10-tetra­aza­cyclo­dodecane (247 mg, 1.4 mmol) and copper(II) perchlorate hexa­hydrate (527 mg, 1.4 mmol) were separately dissolved in 2.8 mL anhydrous methanol each and combined. The resulting purple solution formed a precipitate. The reaction mixture was heated to reflux for 30 min then filtered. The filtrate was evaporated to dryness to yield a purple amorphous solid. X-ray quality crystals were grown by dissolving the solid in a minimum amount of methanol followed by slow evaporation at ambient temperature. The title complex [Cu(cyclen)](ClO_4_)_2_ was isolated as purple crystals in 84% yield (1.2 mmol, 526 mg). IR [ATR, ν (cm^−1^)]: 3281, 2939, 1478, 1072, 617. MS (MALDI–TOF, MeOH): *m*/*z* = 334.2 [Cu(cyclen)^2+^ + ClO_4_^−^]^−^.

## Refinement   

Crystal data, data collection and structure refinement details are summarized in Table 2 ▸. H atoms attached to carbon were positioned geometrically and constrained to ride on their parent atoms. The H atoms attached to nitro­gen were located in a difference map and restrained to have comparable bond lengths. *U*_iso_(H) values were set to 1.2*U*_eq_(C/N).

## Supplementary Material

Crystal structure: contains datablock(s) I. DOI: 10.1107/S2056989016019563/zl2687sup1.cif

Structure factors: contains datablock(s) I. DOI: 10.1107/S2056989016019563/zl2687Isup2.hkl

CCDC reference: 1521075

Additional supporting information: crystallographic information; 3D view; checkCIF report

## Figures and Tables

**Table 1:** Hydrogen-bond geometry (Å, °)

*D*—H⋯*A*	*D*—H	H⋯*A*	*D*⋯*A*	*D*—H⋯*A*
N1—H1⋯O6^i^	0.86 (1)	2.50 (2)	3.171 (1)	135 (1)
N1—H1⋯O2	0.86 (1)	2.39 (1)	3.093 (1)	139 (1)
N2—H2⋯O8^ii^	0.88 (2)	2.31 (2)	3.050 (1)	142 (1)
N2—H2⋯O3	0.88 (2)	2.44 (2)	3.052 (2)	127 (1)
N3—H3⋯O1^ii^	0.86 (2)	2.40 (1)	3.245 (1)	169 (2)
N3—H3⋯O4^ii^	0.86 (2)	2.55 (2)	3.132 (1)	126 (1)
N4—H4⋯O5	0.86 (2)	2.36 (1)	3.096 (1)	143 (1)

Symmetry codes: (i) 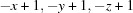; (ii) 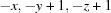.

**Table 2:** Experimental details

Crystal data	
Chemical formula	[Cu(ClO_4_)(C_8_H_20_N_4_)]ClO_4_
*M*_r_	434.72
Crystal system, space group	Monoclinic, *P*2_1_/*n*
Temperature (K)	173
*a*, *b*, *c* (Å)	8.9387 (2), 15.0607 (4), 11.9235 (3)
β (°)	92.949 (1)
*V* (Å^3^)	1603.05 (7)
*Z*	4
Radiation type	Mo *K*α
μ (mm^−1^)	1.74
Crystal size (mm)	0.23 × 0.21 × 0.18

Data collection	
Diffractometer	Bruker SMART APEXII CCD
Absorption correction	Multi-scan (*SADABS*; Sheldrick, 2014 ▸)
*T*_min_, *T*_max_	0.667, 0.747
No. of measured, independent and observed [*I* > 2σ(*I*)] reflections	43306, 7519, 6655
*R*_int_	0.021
(sin θ/λ)_max_ (Å^−1^)	0.830

Refinement	
*R*[*F* ^2^ > 2σ(*F* ^2^)], *wR*(*F* ^2^), *S*	0.025, 0.068, 1.02
No. of reflections	7519
No. of parameters	221
No. of restraints	6
H-atom treatment	H atoms treated by a mixture of independent and constrained refinement
Δρ_max_, Δρ_min_ (e Å^−3^)	0.60, −0.44

Computer programs: *APEX2* and *SAINT-Plus* (Bruker, 2013 ▸), *SHELXT* (Sheldrick, 2008 ▸), *SHELXL2014* (Sheldrick, 2015 ▸) and *SHELXLE* (Hübschle *et al.*, 2011 ▸).

## supplementary crystallographic information

### Crystal data

**Table:**

[Cu(ClO_4_)(C_8_H_20_N_4_)]ClO_4_	*F*(000) = 892
*M**_r_* = 434.72	*D*_x_ = 1.801 Mg m^−^^3^
Monoclinic, *P*2_1_/*n*	Mo *K*α radiation, λ = 0.71073 Å
*a* = 8.9387 (2) Å	Cell parameters from 9899 reflections
*b* = 15.0607 (4) Å	θ = 2.3–35.9°
*c* = 11.9235 (3) Å	µ = 1.74 mm^−^^1^
β = 92.949 (1)°	*T* = 173 K
*V* = 1603.05 (7) Å^3^	Block, purple
*Z* = 4	0.23 × 0.21 × 0.18 mm

### Data collection

**Table:**

Bruker SMART APEXII CCD diffractometer	6655 reflections with *I* > 2σ(*I*)
Radiation source: fine focus sealed tube	*R*_int_ = 0.021
phi and ω scans	θ_max_ = 36.2°, θ_min_ = 2.2°
Absorption correction: multi-scan (SADABS; Sheldrick, 2014)	*h* = −14→13
*T*_min_ = 0.667, *T*_max_ = 0.747	*k* = −24→24
43306 measured reflections	*l* = −19→12
7519 independent reflections	

### Refinement

**Table:**

Refinement on *F*^2^	Secondary atom site location: difference Fourier map
Least-squares matrix: full	Hydrogen site location: mixed
*R*[*F*^2^ > 2σ(*F*^2^)] = 0.025	H atoms treated by a mixture of independent and constrained refinement
*wR*(*F*^2^) = 0.068	*w* = 1/[σ^2^(*F*_o_^2^) + (0.0357*P*)^2^ + 0.4526*P*] where *P* = (*F*_o_^2^ + 2*F*_c_^2^)/3
*S* = 1.02	(Δ/σ)_max_ = 0.003
7519 reflections	Δρ_max_ = 0.60 e Å^−^^3^
221 parameters	Δρ_min_ = −0.44 e Å^−^^3^
6 restraints	Extinction correction: SHELXL2014 (Sheldrick, 2015), Fc^*^=kFc[1+0.001xFc^2^λ^3^/sin(2θ)]^-1/4^
Primary atom site location: structure-invariant direct methods	Extinction coefficient: 0.0025 (3)

### Special details

**Table:**

Geometry. All esds (except the esd in the dihedral angle between two l.s. planes) are estimated using the full covariance matrix. The cell esds are taken into account individually in the estimation of esds in distances, angles and torsion angles; correlations between esds in cell parameters are only used when they are defined by crystal symmetry. An approximate (isotropic) treatment of cell esds is used for estimating esds involving l.s. planes.

### Fractional atomic coordinates and isotropic or equivalent isotropic displacement parameters (Å^2^)

**Table:**

	*x*	*y*	*z*	*U*_iso_*/*U*_eq_	
C1	0.42855 (13)	0.41595 (9)	0.83806 (10)	0.0315 (2)	
H1A	0.5240	0.4277	0.8813	0.038*	
H1B	0.4043	0.3521	0.8450	0.038*	
C2	0.30439 (15)	0.47211 (10)	0.88238 (9)	0.0341 (2)	
H2A	0.2850	0.4540	0.9601	0.041*	
H2B	0.3344	0.5354	0.8835	0.041*	
C3	0.07219 (14)	0.38402 (9)	0.83922 (11)	0.0330 (2)	
H3A	0.0198	0.3977	0.9083	0.040*	
H3B	0.1353	0.3308	0.8533	0.040*	
C4	−0.04050 (12)	0.36717 (8)	0.74254 (13)	0.0353 (3)	
H4A	−0.0945	0.3110	0.7554	0.042*	
H4B	−0.1148	0.4160	0.7378	0.042*	
C5	0.08786 (13)	0.27094 (7)	0.60632 (10)	0.0297 (2)	
H5A	0.0006	0.2334	0.5836	0.036*	
H5B	0.1415	0.2431	0.6720	0.036*	
C6	0.19107 (16)	0.27947 (8)	0.51035 (10)	0.0345 (2)	
H6A	0.2356	0.2209	0.4943	0.041*	
H6B	0.1335	0.2998	0.4420	0.041*	
C7	0.44486 (13)	0.30541 (7)	0.60226 (11)	0.0302 (2)	
H7A	0.5046	0.2707	0.5500	0.036*	
H7B	0.4126	0.2651	0.6620	0.036*	
C8	0.53867 (12)	0.38048 (8)	0.65356 (12)	0.0315 (2)	
H8A	0.6201	0.3558	0.7037	0.038*	
H8B	0.5847	0.4147	0.5934	0.038*	
H1	0.4734 (19)	0.4937 (9)	0.7172 (14)	0.038*	
H2	0.1146 (18)	0.5097 (10)	0.8063 (14)	0.038*	
H3	−0.0155 (19)	0.3802 (12)	0.5781 (13)	0.038*	
H4	0.3389 (19)	0.3719 (11)	0.4820 (12)	0.038*	
N1	0.44205 (10)	0.43993 (6)	0.71871 (8)	0.02310 (15)	
N2	0.16614 (10)	0.46012 (7)	0.80849 (8)	0.02595 (16)	
N3	0.03746 (11)	0.36146 (6)	0.63546 (9)	0.02866 (19)	
N4	0.31208 (12)	0.34444 (6)	0.54087 (8)	0.02774 (18)	
O1	0.19181 (10)	0.55583 (5)	0.55695 (6)	0.02513 (14)	
O2	0.40695 (9)	0.63197 (6)	0.62923 (8)	0.03461 (19)	
O3	0.17927 (13)	0.64843 (8)	0.71488 (9)	0.0461 (3)	
O4	0.21084 (11)	0.71021 (6)	0.53609 (9)	0.0388 (2)	
O5	0.24547 (13)	0.44353 (6)	0.31635 (7)	0.0375 (2)	
O6	0.36978 (11)	0.40926 (8)	0.15317 (8)	0.0381 (2)	
O7	0.21333 (13)	0.30342 (6)	0.23229 (9)	0.0411 (2)	
O8	0.10864 (11)	0.42926 (7)	0.14227 (8)	0.0380 (2)	
Cl1	0.24776 (3)	0.63777 (2)	0.61025 (2)	0.02082 (4)	
Cl2	0.23354 (3)	0.39588 (2)	0.21099 (2)	0.02216 (5)	
Cu1	0.23119 (2)	0.42802 (2)	0.65438 (2)	0.01773 (3)	

### Atomic displacement parameters (Å^2^)

**Table:**

	*U*^11^	*U*^22^	*U*^33^	*U*^12^	*U*^13^	*U*^23^
C1	0.0265 (5)	0.0390 (6)	0.0278 (5)	−0.0022 (4)	−0.0100 (4)	0.0095 (4)
C2	0.0368 (6)	0.0465 (7)	0.0184 (4)	−0.0037 (5)	−0.0038 (4)	−0.0015 (4)
C3	0.0296 (5)	0.0328 (5)	0.0375 (6)	0.0004 (4)	0.0110 (4)	0.0077 (4)
C4	0.0176 (4)	0.0285 (5)	0.0600 (8)	−0.0005 (4)	0.0049 (5)	0.0049 (5)
C5	0.0299 (5)	0.0191 (4)	0.0386 (5)	−0.0049 (3)	−0.0117 (4)	0.0052 (4)
C6	0.0523 (7)	0.0237 (5)	0.0266 (5)	−0.0087 (5)	−0.0064 (5)	−0.0022 (4)
C7	0.0307 (5)	0.0206 (4)	0.0402 (6)	0.0008 (4)	0.0099 (4)	0.0013 (4)
C8	0.0199 (4)	0.0252 (5)	0.0501 (7)	−0.0006 (3)	0.0081 (4)	0.0014 (4)
N1	0.0190 (3)	0.0217 (3)	0.0282 (4)	−0.0023 (3)	−0.0021 (3)	0.0033 (3)
N2	0.0241 (4)	0.0280 (4)	0.0259 (4)	0.0011 (3)	0.0030 (3)	0.0013 (3)
N3	0.0236 (4)	0.0208 (4)	0.0400 (5)	−0.0022 (3)	−0.0134 (3)	0.0065 (3)
N4	0.0403 (5)	0.0208 (4)	0.0224 (4)	−0.0040 (3)	0.0032 (3)	0.0018 (3)
O1	0.0361 (4)	0.0155 (3)	0.0227 (3)	−0.0025 (3)	−0.0091 (3)	−0.0012 (2)
O2	0.0213 (4)	0.0348 (4)	0.0466 (5)	−0.0019 (3)	−0.0095 (3)	0.0080 (4)
O3	0.0542 (6)	0.0452 (6)	0.0408 (5)	−0.0136 (5)	0.0192 (4)	−0.0216 (4)
O4	0.0420 (5)	0.0181 (3)	0.0538 (5)	−0.0016 (3)	−0.0219 (4)	0.0084 (3)
O5	0.0614 (6)	0.0281 (4)	0.0222 (3)	0.0054 (4)	−0.0054 (4)	−0.0058 (3)
O6	0.0258 (4)	0.0516 (6)	0.0371 (4)	−0.0021 (4)	0.0033 (3)	0.0019 (4)
O7	0.0536 (6)	0.0203 (4)	0.0493 (5)	−0.0032 (4)	0.0031 (5)	−0.0015 (4)
O8	0.0302 (4)	0.0538 (6)	0.0295 (4)	0.0164 (4)	−0.0042 (3)	−0.0005 (4)
Cl1	0.02111 (9)	0.01695 (8)	0.02384 (9)	−0.00122 (7)	−0.00426 (7)	−0.00201 (7)
Cl2	0.02481 (10)	0.02058 (9)	0.02081 (9)	0.00237 (7)	−0.00149 (7)	−0.00166 (7)
Cu1	0.01806 (5)	0.01641 (5)	0.01824 (5)	−0.00136 (3)	−0.00382 (4)	0.00267 (3)

### Geometric parameters (Å, º)

**Table:**

C1—N1	1.4791 (14)	C7—H7A	0.9900
C1—C2	1.5122 (19)	C7—H7B	0.9900
C1—H1A	0.9900	C8—N1	1.4901 (15)
C1—H1B	0.9900	C8—H8A	0.9900
C2—N2	1.4913 (15)	C8—H8B	0.9900
C2—H2A	0.9900	N1—Cu1	2.0061 (9)
C2—H2B	0.9900	N1—H1	0.858 (14)
C3—N2	1.4781 (16)	N2—Cu1	2.0145 (9)
C3—C4	1.513 (2)	N2—H2	0.876 (14)
C3—H3A	0.9900	N3—Cu1	2.0036 (9)
C3—H3B	0.9900	N3—H3	0.859 (13)
C4—N3	1.4881 (18)	N4—Cu1	2.0099 (10)
C4—H4A	0.9900	N4—H4	0.859 (13)
C4—H4B	0.9900	O1—Cl1	1.4644 (7)
C5—N3	1.4826 (15)	O1—Cu1	2.2664 (7)
C5—C6	1.5118 (19)	O2—Cl1	1.4320 (8)
C5—H5A	0.9900	O3—Cl1	1.4267 (10)
C5—H5B	0.9900	O4—Cl1	1.4321 (9)
C6—N4	1.4898 (15)	O5—Cl2	1.4459 (9)
C6—H6A	0.9900	O6—Cl2	1.4441 (10)
C6—H6B	0.9900	O7—Cl2	1.4285 (9)
C7—N4	1.4835 (16)	O8—Cl2	1.4409 (9)
C7—C8	1.5174 (17)		
			
N1—C1—C2	107.30 (9)	C1—N1—C8	115.66 (9)
N1—C1—H1A	110.3	C1—N1—Cu1	102.97 (7)
C2—C1—H1A	110.3	C8—N1—Cu1	107.77 (7)
N1—C1—H1B	110.3	C1—N1—H1	107.1 (11)
C2—C1—H1B	110.3	C8—N1—H1	110.9 (12)
H1A—C1—H1B	108.5	Cu1—N1—H1	112.2 (11)
N2—C2—C1	109.06 (9)	C3—N2—C2	114.31 (10)
N2—C2—H2A	109.9	C3—N2—Cu1	103.55 (7)
C1—C2—H2A	109.9	C2—N2—Cu1	107.38 (7)
N2—C2—H2B	109.9	C3—N2—H2	111.3 (12)
C1—C2—H2B	109.9	C2—N2—H2	109.4 (12)
H2A—C2—H2B	108.3	Cu1—N2—H2	110.7 (11)
N2—C3—C4	107.81 (10)	C5—N3—C4	114.55 (9)
N2—C3—H3A	110.1	C5—N3—Cu1	102.42 (7)
C4—C3—H3A	110.1	C4—N3—Cu1	108.34 (7)
N2—C3—H3B	110.1	C5—N3—H3	106.1 (12)
C4—C3—H3B	110.1	C4—N3—H3	113.6 (12)
H3A—C3—H3B	108.5	Cu1—N3—H3	111.3 (12)
N3—C4—C3	109.92 (9)	C7—N4—C6	114.37 (9)
N3—C4—H4A	109.7	C7—N4—Cu1	102.84 (7)
C3—C4—H4A	109.7	C6—N4—Cu1	107.10 (8)
N3—C4—H4B	109.7	C7—N4—H4	110.1 (12)
C3—C4—H4B	109.7	C6—N4—H4	110.2 (12)
H4A—C4—H4B	108.2	Cu1—N4—H4	112.0 (12)
N3—C5—C6	107.69 (9)	Cl1—O1—Cu1	116.92 (4)
N3—C5—H5A	110.2	O3—Cl1—O2	109.65 (7)
C6—C5—H5A	110.2	O3—Cl1—O4	111.02 (7)
N3—C5—H5B	110.2	O2—Cl1—O4	109.85 (6)
C6—C5—H5B	110.2	O3—Cl1—O1	108.78 (6)
H5A—C5—H5B	108.5	O2—Cl1—O1	109.36 (5)
N4—C6—C5	109.55 (9)	O4—Cl1—O1	108.14 (5)
N4—C6—H6A	109.8	O7—Cl2—O8	109.87 (7)
C5—C6—H6A	109.8	O7—Cl2—O6	109.78 (7)
N4—C6—H6B	109.8	O8—Cl2—O6	109.12 (6)
C5—C6—H6B	109.8	O7—Cl2—O5	109.49 (6)
H6A—C6—H6B	108.2	O8—Cl2—O5	109.95 (6)
N4—C7—C8	108.37 (9)	O6—Cl2—O5	108.61 (6)
N4—C7—H7A	110.0	N3—Cu1—N1	151.33 (4)
C8—C7—H7A	110.0	N3—Cu1—N4	87.11 (4)
N4—C7—H7B	110.0	N1—Cu1—N4	87.14 (4)
C8—C7—H7B	110.0	N3—Cu1—N2	86.24 (4)
H7A—C7—H7B	108.4	N1—Cu1—N2	86.52 (4)
N1—C8—C7	109.56 (9)	N4—Cu1—N2	153.54 (4)
N1—C8—H8A	109.8	N3—Cu1—O1	104.87 (3)
C7—C8—H8A	109.8	N1—Cu1—O1	103.78 (3)
N1—C8—H8B	109.8	N4—Cu1—O1	103.79 (3)
C7—C8—H8B	109.8	N2—Cu1—O1	102.66 (3)
H8A—C8—H8B	108.2		
			
N1—C1—C2—N2	54.05 (13)	C6—C5—N3—C4	−168.48 (9)
N2—C3—C4—N3	50.52 (13)	C6—C5—N3—Cu1	−51.42 (9)
N3—C5—C6—N4	53.24 (12)	C3—C4—N3—C5	89.53 (11)
N4—C7—C8—N1	51.48 (13)	C3—C4—N3—Cu1	−24.09 (11)
C2—C1—N1—C8	−169.09 (9)	C8—C7—N4—C6	−165.51 (10)
C2—C1—N1—Cu1	−51.82 (10)	C8—C7—N4—Cu1	−49.77 (10)
C7—C8—N1—C1	89.34 (12)	C5—C6—N4—C7	87.24 (12)
C7—C8—N1—Cu1	−25.21 (11)	C5—C6—N4—Cu1	−26.00 (11)
C4—C3—N2—C2	−166.49 (10)	Cu1—O1—Cl1—O3	−58.89 (8)
C4—C3—N2—Cu1	−50.00 (10)	Cu1—O1—Cl1—O2	60.85 (7)
C1—C2—N2—C3	87.01 (12)	Cu1—O1—Cl1—O4	−179.55 (6)
C1—C2—N2—Cu1	−27.24 (11)		

### Hydrogen-bond geometry (Å, º)

**Table:**

*D*—H···*A*	*D*—H	H···*A*	*D*···*A*	*D*—H···*A*
N1—H1···O6^i^	0.86 (1)	2.50 (2)	3.171 (1)	135 (1)
N1—H1···O2	0.86 (1)	2.39 (1)	3.093 (1)	139 (1)
N2—H2···O8^ii^	0.88 (2)	2.31 (2)	3.050 (1)	142 (1)
N2—H2···O3	0.88 (2)	2.44 (2)	3.052 (2)	127 (1)
N3—H3···O1^ii^	0.86 (2)	2.40 (1)	3.245 (1)	169 (2)
N3—H3···O4^ii^	0.86 (2)	2.55 (2)	3.132 (1)	126 (1)
N4—H4···O5	0.86 (2)	2.36 (1)	3.096 (1)	143 (1)

Symmetry codes: (i) −*x*+1, −*y*+1, −*z*+1; (ii) −*x*, −*y*+1, −*z*+1.
